# The Preliminary Training School for Nurses. II. Some Early and Later Experiences
*The first article was published in The Hospital of May 26, p. 155.


**Published:** 1917-06-02

**Authors:** 


### THE MATRONS' AND SISTERS' DEPARTMENT.

### THE PRELIMINARY TRAINING SCHOOL FOR NURSES.*

### II. Some Early and Later Experiences.

MORE than twenty-two years ago, Miss Luckes, the matron of the London Hospital, realised that it was impossible to get the best results from beginning the training of probationers in hospital wards. Much time was wasted because even those who were very keen were handicapped in their training by those in charge being too busy to give enough time and attention to new-comers.

After a good deal of discussion as to cost of up-keep and advantages to be gained, a house was taken in Bow Road, bathrooms were arranged for, and some of the larger bedrooms were made into cubicles. A greenhouse was turned into a lecture-room and a stable into a sitting-room, and the whole place was made as suitable as possible for the purpose. Twenty selected candidates entered on a given date for seven weeks' preliminary training. Later on, a small house next door to this becoming empty, was taken and altered; the small kitchen was turned into a bathroom, and the other rooms were divided to make room for more probationers; a passage in the basement connected the two houses. This allowed another eight probationers to be taken, thus bringing the number up to twenty-eight probationers received at one time.

In 1910 the late Lord Tredegar kindly gave the London Hospital the freehold of these two houses; so as they were very old, and in many ways inconvenient, it was decided to pull them down and build a quite new house on the same site, which would combine the comforts of a home with the necessary lecture-rooms and requirements of an institution. A large building in the neighbourhood was taken temporarily so that the preliminary training was not interrupted. The new building was completed in the spring of 1912, the whole cost of the building and furnishing being provided by those who had been nursed by nurses trained at the London Hospital, and by private benefactors; none of the money being taken from hospital funds.

This house contains bed and sitting rooms for three sisters, separate bedrooms for thirty-two probationers, three maids' bedrooms and their sitting-room; ten bathrooms, fitted with hot pipes for drying towels; a large dining-room, with servery opening out of it, to which all food is brought from the kitchen by a lift; a large pleasant sitting-room with easy-chairs and a piano; a lecture-room and two small class-rooms, one of which is fitted up as a small ward, and the bed contains the dummy patient. There is a convenient cloak-room with fixed basins for washing hands, besides all the usual domestic offices, an electric passenger lift, and the house is well heated and ventilated.

The garden is large and contains two summer-houses, a tennis court and croquet lawn, also a small vegetable garden. There is a good kitchen, working pantry, etc., in a light basement, but these are entirely for the use of the domestic staff. Thirty-two candidates are now received every seven weeks during the year, except when the school is closed for holidays.

The candidates arrive on a Saturday afternoon, as this gives them a quiet day on Sunday to settle down; they begin to come about 4 P.M., and by 7 P.M. the number is generally complete. It is quite possible to get a good idea of the sort of probationers they are likely to be from their attitude on arrival. Some are quite ordinary, just come in and are introduced by the maid, are taken to their bedrooms by a sister, unpack, and get into uniform and settle down almost at once. Others arrive in tears and have to be first comforted! Others arrive with friends, who affect the probationer in various ways; some brace her up, and others appear to do their utmost to upset her; some want to wait and see her in uniform. These friends are shown round and given tea if they come from a distance, and all the probationers are given tea as they arrive.

By 7.30 P.M. most will have unpacked and be in uniform; they are then shown how to make up and wear their caps and how to do their hair. Supper the first night is at 8 P.M., as it always takes longer than the usual half-hour. Then the probationers are divided into two parties and shown round the house, the lift and the fire appliances are explained, and any questions are answered. By 9.30 P.M. they are free to go to bed or to their sitting-room, where someone generally plays the piano, and by 10 P.M. they all retire to their bedrooms. The next day being Sunday, breakfast is not until 9 A.M., so they have time to rest after their journeys. After breakfast each one is told which part of the housework she will do; some wash up the breakfast-things, others do the dining, sitting, and breakfast rooms, while others make beds, clean baths, sweep and dust, etc.; the work of the house, with the exception of the sisters' rooms, and any scrubbing or fireplaces, being done by the probationers. (This work takes about an hour and is changed every fortnight, so that each one is shown each part of the work.) The rest of the Sunday, with the exception of a class in the afternoon, when time-tables, etc., are explained, is practically free; the probationers generally attend the hospital church in the evening.

*(To be continued.)*

* The first article was published in THE HOSPITAL of May 26, p. 155.

